# Extrusion Processing and Formulation Effects on the Techno‐Functional Properties of Melon Peel‐Enriched Soy Protein–Maize Starch Snacks

**DOI:** 10.1155/ijfo/4987343

**Published:** 2026-06-24

**Authors:** Betsabé Hernández-Santos, Jesús Rodríguez-Miranda, Erasmo Herman-Lara, Emmanuel J. Ramírez-Rivera, Juan G. Torruco-Uco, José M. Juárez-Barrientos, José M. Rivadeneyra-Rodríguez, Andrea Ángeles-Hernández, Enrique Ramírez-Figueroa

**Affiliations:** ^1^ Department of Chemical and Biochemical Engineering, National Technological of Mexico/Technological Institute of Tuxtepec, San Juan Bautista Tuxtepec, Oaxaca, Mexico; ^2^ Engineering in Sustainable Agricultural Innovation, National Technological of Mexico/Higher Technological Institute of Zongolica, Zongolica, Veracruz, Mexico; ^3^ Institute of Agricultural Engineering, University of Papaloapan, Loma Bonita Campus, Loma Bonita Oax, Mexico, unpa.edu.mx

**Keywords:** extrusion cooking, functional snacks, melon peel, response surface methodology

## Abstract

The valorisation of agro‐industrial by‐products through extrusion processing represents a sustainable strategy for developing value‐added foods. This study evaluated the effects of barrel temperature (120°C–170°C), feed moisture content (16–25 g/100 g), soy protein concentrate/maize starch ratio (25–40 g/100 g) and melon peel flour content (10–25 g/100 g) on the process parameters and techno‐functional properties of extruded snacks. A central composite design and response surface methodology were applied to model residence time (RT), torque (TO), specific mechanical energy (SME), expansion index (EI), apparent density (AD), hardness (HA), water absorption index (WAI), water solubility index (WSI), pH and total colour difference (*Δ*E). RT, EI, AD, WAI, WSI, pH and *Δ*E were adequately described by significant models (*p* < 0.05), with coefficients of determination ranging from 0.72 to 0.98. Feed moisture content was the main factor affecting extrusion performance, reducing SME and expansion while increasing density and HA. In contrast, higher melon peel levels promoted denser and mechanically stronger structures due to the incorporation of dietary fibre. Experimental values ranged from 26.12 to 35.96 s for RT, 1.62 to 2.12 for EI, 0.30 to 0.52 g/cm^3^ for AD and 18.95 to 36.09 N for HA. The results demonstrate that fibre–protein–starch interactions strongly influence melt rheology and product structure. These findings confirm the technological feasibility of incorporating melon peel flour into extrusion‐based snacks and provide new insights into the role of dietary fibre in starch–protein extrusion systems.

## 1. Introduction

The growing interest in the development of functional and sustainable foods has stimulated the search for alternative ingredients capable of enhancing the nutritional value of food products while simultaneously contributing to the reduction of food waste. In this context, the valorisation of agro‐industrial by‐products has gained increasing importance within the framework of the circular economy, as it enables residues rich in bioactive compounds to be transformed into functional ingredients for the food industry [1, 2]. Various fruit and vegetable by‐products contain significant amounts of dietary fibre, phenolic compounds, vitamins and minerals, making them promising raw materials for the development of value‐added foods.

Among these by‐products, melon peel (MP) (*Cucumis melo* L.) has attracted attention due to its high dietary fibre content, structural polysaccharides and phenolic compounds with antioxidant activity. Recent studies have shown that residues generated during melon processing may contain considerable concentrations of bioactive compounds capable of improving the functional and nutritional properties of foods while also contributing to reducing the environmental impact associated with fruit waste [2]. Consequently, the incorporation of such by‐products into food matrices represents a viable strategy for the development of innovative and sustainable products. MP has been widely reported as a source of dietary fibre and phenolic compounds with antioxidant potential, supporting its relevance as a promising ingredient for fibre‐enriched extrusion‐based formulations [3].

In parallel, extrusion has become one of the most versatile and efficient technologies for producing expanded snacks and ready‐to‐eat foods. This process involves the combined action of high temperature, pressure and shear forces, which induce important physicochemical transformations in the ingredients, including starch gelatinisation, protein denaturation and the formation of porous structures characteristic of extruded products [4]. These transformations determine key properties of the final product, such as expansion index (EI), apparent density (AD), texture and hydration characteristics.

Conventional extruded snacks are commonly formulated using starch‐rich raw materials such as maize, rice or wheat flours, which provide the structural basis required for expansion during extrusion cooking [1]. In many formulations, these cereal matrices are combined with plant protein ingredients, such as soy protein concentrates (SPCs), to improve the nutritional profile of the final product. However, the incorporation of nonstarch components, particularly protein‐rich ingredients and dietary fibre from agro‐industrial by‐products, may significantly alter melt rheology and reduce expansion capacity due to dilution of the continuous starch phase and interference with bubble growth during die exit [4]. These effects frequently result in products with higher AD and hardness (HA), which represent important technological challenges in the development of expanded extruded snacks enriched with sustainable ingredients.

However, the incorporation of fibre‐rich ingredients or plant by‐products can significantly modify the behaviour of the system during extrusion. In particular, dietary fibre may interfere with starch gelatinisation and the development of the cellular structure of the extrudate, generally leading to a reduction in EI and an increase in density and HA of the product [5, 6]. For this reason, the development of extruded foods enriched with by‐products requires an appropriate balance between formulation and processing conditions in order to ensure acceptable technological quality.

To address this complexity, response surface methodology (RSM) has become a widely used statistical tool for the optimisation of extrusion processes. This methodology enables the simultaneous evaluation of multiple process and formulation variables, as well as their interactions, thereby facilitating the identification of optimal conditions that maximise the technological and functional properties of the product [6]. Several studies have successfully applied RSM to optimise extruded formulations incorporating plant proteins, agro‐industrial by‐products and different starch sources, improving both structural quality and the nutritional value of the final products [7].

Despite the growing interest in the valorisation of fruit by‐products through extrusion technologies, information regarding the use of MP as a functional ingredient in extruded snacks remains limited, and there is scarce evidence on how the interaction between MP content and extrusion conditions simultaneously affects the process behaviour and the physical and functional properties of the final product. Although previous RSM‐based extrusion studies have addressed the incorporation of various fruit and vegetable by‐products (e.g., mango peel, apple pomace, pumpkin seed), to our knowledge the combined effects of MP flour, soy protein concentrate/maize starch ratio (SPC/MS), barrel temperature and feed moisture content on both process variables and techno‐functional properties have not been systematically evaluated.

The novelty of the present study lies in the mechanistic characterisation of fibre–protein–starch interactions under controlled thermomechanical conditions, providing new insight into how insoluble fibre fractions from MP modulate melt rheology, expansion behaviour and hydration properties in a starch–protein extrusion system. Therefore, the present study is aimed at investigating the combined influence of barrel temperature, feed moisture content, SPC/MS ratio and MP level on the techno‐functional properties of extruded snacks, contributing to the development of sustainable extrusion‐based formulations within a circular economy framework.

## 2. Materials and Methods

Melons were purchased from a local market in San Juan Bautista Tuxtepec, Oaxaca, Mexico. SPC was obtained from the commercial brand FTT (FUDTECH, Food Technologies Trading S.A. de C.V., Mexico), whereas maize starch (MS) was acquired from IMSA (Industrializadora de Maíz S.A. de C.V., Tlalnepantla de Baz, State of Mexico, Mexico).

### 2.1. Preparation of MP Flour

The melons were washed and peeled following the methodology described by Téllez‐Morales et al. [8]. The peels were cut into fragments of approximately 1 cm and subsequently dried in a tray dryer at 60°C for 8 h. This drying protocol was selected to minimise potential degradation of functional components (dietary fibre, phenolic compounds) while achieving adequate moisture reduction, in accordance with conditions reported for MP valorisation [2, 9]. The yield of MP flour was approximately 12.5 g per 100 g of fresh peel (wet basis), consistent with the moisture reduction achieved during drying and milling. The dried material was then ground using a domestic grinder (KRUPS, model GX410011, Mexico) and sieved to obtain a particle size of 0.59 mm aperture (No. 30, U.S. standard sieve).

### 2.2. Proximate Chemical Analysis of Raw Materials

The chemical composition of the raw materials was determined according to the official AOAC methods [10], including moisture (925.10), ash (923.03), protein (920.87), fat (920.39) and crude fibre (962.09). Total carbohydrate content was calculated by difference. All results are expressed on a wet basis (as‐is). All determinations were performed in triplicate.

### 2.3. Preparation of the Mixtures

The raw materials were blended according to the proportions defined by the experimental design (Table 1). The mixtures were conditioned to different moisture contents by the addition of distilled water, which was incorporated manually. Subsequently, the samples were placed in sealed plastic bags and stored at 4°C for 12 h to ensure homogeneous water distribution within the matrix prior to the extrusion process. This conditioning period was selected on the basis of Leyva‐Corral et al. [11], who reported that a minimum of 12 h at 4°C is required to achieve uniform hydration in starch–protein blends intended for extrusion, thereby preventing moisture gradients that could affect process stability and product quality.

**Table 1:** Process variables and levels used in the experimental design.

Variables	Levels				
	−2	−1	0	+1	+2
Barrel temperature (BT: °C)	120	132.50	145	145.00	170
Moisture content (MC: g/100 g)	16	18.25	20.50	22.75	25
Soy protein concentrate/maize starch (SPC/MS: g/100 g)	25	28.75	32.50	36.25	40
Melon peel (MP: g/100 g)	10	13.75	17.50	21.25	25

### 2.4. Extrusion Process

Extrusion was carried out using a laboratory‐scale single‐screw extruder (Extruder 19/25DN, model 832005.007, Brabender GmbH & Co. KG, Germany), with a barrel length of 428 mm, a screw diameter of 19 mm and a compression ratio of 3:1. A circular die with an internal diameter of 4 mm was used. The barrel temperature profile was set at 50°C (Zone 1), 80°C (Zone 2), 100°C (Zone 3) and 120°C–170°C in Zone 4, corresponding to the die section (Table 1). The feed rate was maintained at 60 g/min, and the screw speed was set at 150 rpm.

The extrusion conditions were selected based on previous studies involving starch–protein extrusion systems and fibre‐enriched formulations processed using laboratory‐scale single‐screw extruders. The selected barrel temperature profile was designed to promote progressive starch gelatinisation, protein denaturation and melt formation while avoiding excessive thermal degradation. Likewise, the screw speed (150 rpm) and feed rate (60 g/min) were chosen to provide stable material flow, adequate shear generation and sufficient residence time (RT) for thermomechanical transformation of the mixture. Similar extrusion conditions have been successfully applied in the production of expanded snacks formulated with cereal starches, plant proteins and agro‐industrial by‐products [12–14].

### 2.5. Process Variables Evaluated

#### 2.5.1. Determination of RT, Torque (TO) and Specific Mechanical Energy (SME)

RT, TO and SME were determined according to the methodology reported by Pensamiento‐Niño et al. [12]. RT was measured by injecting a tracer (FD&C Red No. 40 dye) at the feed port and recording the time elapsed until its appearance at the die exit. TO was recorded directly from the extruder TO display. SME was calculated according to the following equation: $\text{SME}\;\left(\text{J}/\text{g}\right)=\left[2\pi \times \text{N}\times \text{T}\times \left(\text{MC}/100\right)\right]/\dot{\text{Q}}$, where N is the screw speed (rev/s), T is the TO (N·m), MC is the percentage of maximum TO used (%) and Q̇ is the mass flow rate (kg/s) [12].

### 2.6. Characterisation of the Extruded Product

#### 2.6.1. Determination of EI and AD

The EI was determined by measuring the diameter of 25 extrudates using a digital vernier caliper (Science Purchase, model 0604CAL6, United States) and dividing the average extrudate diameter by the internal diameter of the extruder die (4 mm) [15]. The AD was determined by the seed displacement method described by Navarro‐Cortez et al. [16], in which extrudate samples of known mass were immersed in millet seeds within a graduated cylinder, and the volume of seeds displaced was used to calculate density as mass divided by volume (g/cm^3^).

#### 2.6.2. Determination of HA

The HA was determined by measuring the maximum force required to fracture extruded samples of 70 mm in length using a universal texture analyser (Texture Analyzer TA‐XT Plus, Stable Micro Systems, United Kingdom). A Warner–Bratzler blade was employed, and 17 replicates were performed for each treatment [11].

#### 2.6.3. Determination of Water Absorption Index (WAI) and Water Solubility Index (WSI)

WAI and WSI were determined according to the methodology reported by Hernández‐Santos et al. [17]. Briefly, 2.5 g of ground extrudate were dispersed in 30 mL of distilled water at 25°C for 30 min with constant stirring, then centrifuged at 3000 × g for 10 min. The supernatant was decanted and dried at 105°C to constant weight. WAI was calculated as the mass of the wet sediment divided by the initial dry sample mass (g/g), and WSI as the mass of dry solids in the supernatant divided by the initial dry sample mass, expressed as a percentage.

#### 2.6.4. Determination of pH

The pH was measured by mixing 1 g of sample with 10 mL of distilled water at 25°C. Measurements were carried out using a pH metre (Thermo Scientific, Orion Star A211) previously calibrated with buffer solutions at pH 4, 7 and 10.

#### 2.6.5. Evaluation of Total Colour Difference (ΔE)

Colour was determined using a HunterLab tristimulus colourimeter (MiniScan HunterLab, model 45/0 L, Hunter Associates Laboratory Inc., Reston, Virginia, United States). The parameters L* (lightness), a* (red–green chromaticity) and b* (yellow–blue chromaticity) were recorded, and the ΔE was calculated according to Hernández‐Santos et al. [17].

### 2.7. Experimental Design and Data Analysis

A central composite design was employed within the framework of RSM (Tables 1 and 2), using Design‐Expert statistical software Version 7.0.0 (Stat‐Ease Inc., Minneapolis, Minnesota, United States). The independent variables considered were barrel temperature (BT: 120°C–170°C), feed moisture content (MC: 16–25 g/100 g), SPC/MS(SPC/MS: 25–40 g/100 g) and MP content (MP: 10–25 g/100 g). The response variables evaluated were RT, TO, SME, EI, AD, HA, WAI, WSI, pH and ΔE. The statistical significance of the model terms was assessed by analysis of variance (ANOVA) for each response variable. Model adequacy was evaluated through the coefficient of determination (*R* ^2^), and the lack‐of‐fit test (*p* > 0.05 indicates adequate fit). Model validation was performed by comparing predicted versus actual values for each response variable; the resulting plots and adequacy statistics are presented in Tables 3 and 4.

**Table 2:** Chemical composition (g/100 g) of melon peel (MP), maize starch (MS) and soy protein concentrate (SPC).

	MP	SPC	MS
Moisture	10.16 ± 0.09^b^	8.17 ± 0.08^a^	11.39 ± 0.00^c^
Ash	8.76 ± 0.05^c^	5.86 ± 0.16^b^	0.20 ± 0.04^a^
Fat	1.27 ± 0.02^b^	0.34 ± 0.01^a^	0.30 ± 0.00^a^
Protein	3.21 ± 0.00^c^	37.92 ± 0.00^b^	0.88 ± 0.00^a^
Fibre	21.05 ± 0.32^a^	N. D	N. D
Carbohydrates	55.54 ± 0.43^b^	47.71 ± 0.10^a^	87.23 ± 0.04^c^

*Note:* The values represent the average of three determinations ± standard deviation. Different letters in the same row indicate a significant difference (*p* < 0.05).

Abbreviation: N. D., not detected.

**Table 3:** Analysis of variance of the independent and dependent variables of extruded snacks.

Source	DF	Mean squares									
		RT	TO	SME	EI	AD	HA	WAI	WSI	pH	*Δ*E
Model	14	0.069^a^	0.178^a^	4.850^a^	0.006^a^	0.004^a^	0.197^a^	0.027	1.020	0.061	0.014
A‐BT	1	0.079	0.049^a^	7.570^a^	0.029^a^	0.001^a^	0.691^a^	0.032	0.014	0.001	0.001
B‐MC	1	0.310^a^	1.460^a^	19.930^a^	0.038^a^	0.035^a^	0.047	0.053	2.570^a^	0.000	0.038
C‐SPC/MS	1	0.131^a^	0.623^a^	12.470^a^	0.000	0.000	0.298	0.047	2.710^a^	0.006	0.048^a^
D‐MP	1	0.082	0.185^a^	16.190^a^	0.015^a^	0.010^a^	0.034	0.010	2.020	0.008^a^	0.010
AB	1	0.003	0.022	0.922	0.001	0.000	0.000	0.003	0.359	0.000	0.003
AC	1	0.001	0.000	0.165	0.000	0.001	0.352^a^	0.013	0.342	0.000	0.010
AD	1	0.001	0.018	0.302	0.001	0.003	0.399^a^	0.028	0.015	0.000	0.000
BC	1	0.016	0.045^a^	2.150	0.002^a^	0.004	0.481^a^	0.004	0.468	0.000	0.001
BD	1	0.036	0.006	5.250^a^	0.002	0.001^a^	0.001	0.001	1.080	0.000	0.012
CD	1	0.116^a^	0.027	1.360	0.001	0.001^a^	0.016	0.038	2.020	0.000	0.006
*A* ^2^	1	0.077	0.009	0.133	0.001	0.004^a^	0.196	0.029	0.545	0.000	0.018
*B* ^2^	1	0.127^a^	0.045^a^	0.440	0.000	0.001	0.113	0.054	1.240	0.040^a^	0.050^a^
*C* ^2^	1	0.002	0.001	0.009	0.001	0.002^a^	0.180	0.039	0.434	0.006	0.004
*D* ^2^	1	0.008	0.002	0.888	0.001	0.000	0.138	0.016	0.099	0.000	0.007
Residual	15	0.019	0.006	0.897	0.004	0.002	0.071	0.016	0.530	0.022	0.009
Lack of fit	10	0.021	0.006	1.230	0.004	0.001	0.081	0.019	0.751	0.019	0.012
Pure error	5	0.016	0.006	0.223	0.003	0.004	0.052	0.010	0.089	0.003	0.003
*R* ^2^		0.77	0.96	0.83	0.94	0.94	0.72	0.62	0.64	0.73	0.60

Abbreviations: *Δ*E, total colour difference; AD, apparent density; BT, barrel temperature; DF, degrees of freedom; EI, expansion index; HA, hardness; MC, moisture content; MP, melon peel; SPC, soy protein concentrate; MS, maize starch; RT, residence time; SME, specific mechanical energy; TO, torque; WAI, water adsorption index; WSI, water solubility index.

^a^Significance at *p* < 0.05.

**Table 4:** Regression coefficients from a second‐order model of relationships between response and independent variables for extruded snack.

Coe	RT	TO	SME	EI	AD	HA	WAI	WSI	PH	*Δ*E
*b_0_*	5.60^a^	3.24^a^	20.69^a^	1.37^a^	0.628^a^	4.91^a^	1.99	5.45	2.57	6.14
*b_1_*	0.057	−0.045^a^	−0.562^a^	−0.035^a^	−0.008^a^	−0.170^a^	0.037	−0.024	−0.005	0.005
*b_2_*	−0.114^a^	−0.247^a^	−0.911^a^	−0.040^a^	0.038^a^	−0.044	0.047	−0.328^a^	0.002	−0.039
*b_3_*	0.074^a^	0.161^a^	0.721^a^	0.000	−0.004	0.111	−0.044	−0.336^a^	0.016	0.045^a^
*b_4_*	0.058	−0.088^a^	−0.821^a^	−0.025^a^	0.020^a^	−0.037	0.021	−0.290	−0.018	0.021
*b_12_*	0.015	0.037	0.240	−0.009	0.004	−0.001	−0.014	−0.150	0.003	−0.013
*b_13_*	−0.003	0.001	0.101	0.000	0.003	−0.148^a^	−0.029	−0.146	0.005	−0.025
*b_14_*	0.007	0.033	−0.137	0.003	0.004	−0.158^a^	−0.042	−0.030	0.001	−0.002
*b_23_*	−0.032	−0.053^a^	−0.366	0.011^a^	−0.005	−0.173^a^	0.017	0.171	0.005	−0.008
*b_24_*	−0.048	0.019	0.573^a^	0.003	−0.009^a^	−0.009	−0.006	0.260	−0.003	−0.027
*b_34_*	−0.085^a^	−0.041	−0.291	−0.007	0.010^a^	0.032	−0.049	0.355	0.000	0.019
*b_11_*	−0.053	−0.018	0.069	−0.007	0.012^a^	0.085	0.033	−0.141	0.000	0.027
*b_22_*	−0.068^a^	−0.041^a^	−0.127	−0.001	0.002	0.064	0.045	−0.212	−0.038	0.043^a^
*b_33_*	0.009	0.006	0.018	−0.002	0.009^a^	0.081	−0.038	0.126	−0.015	0.012
*b_44_*	−0.017	0.009	0.179	−0.006	−0.000	0.071	0.024	0.060	0.002	0.016

Abbreviations: *Δ*E, total colour difference; Coe, coefficients; EI, expansion index; HA, hardness; RT, residence time; SME, specific mechanical energy; TO, torque; WAI, water adsorption index; WSI, water solubility index.

^a^ Significance at *p* < 0.05.

## 3. Results and Discussion

The chemical composition of the raw materials (Table 2) showed statistically significant differences (*p* < 0.05), which play a key role in determining their behaviour during extrusion processing and their contribution to the structural development of the final product. MP exhibited a high crude fibre content (21.05 g/100 g) and ash content (8.76 g/100 g), together with low protein content (3.21 g/100 g) and a moderate carbohydrate fraction (55.54 g/100 g), confirming its nature as a fibre‐rich agro‐industrial by‐product mainly composed of structural polysaccharides and mineral components. These characteristics are particularly relevant during extrusion, since insoluble fibre fractions may interfere with starch gelatinisation, reduce melt elasticity and limit bubble growth at the die exit, thereby affecting expansion behaviour and increasing product density and HA. This behaviour is consistent with that reported by Gómez‐García et al. [18], who indicated that by‐products of *C. melo* L. contain significant fractions of dietary fibre and minerals associated with the plant structural matrix. Likewise, recent studies on MP flour have documented fibre contents in the range of approximately 18%–25% and ash levels between 6%–9%, which are attributed to the concentration of structural components during drying and milling processes and explain their technological impact when incorporated into extruded systems [19].

Although detailed characterisation of dietary fibre fractions and antioxidant compounds was beyond the scope of the present study, MP has been widely reported as a relevant source of structural polysaccharides such as cellulose, hemicelluloses and pectins, as well as phenolic compounds with recognised antioxidant activity. These components are known to influence hydration behaviour and structural properties in extrusion systems and support the technological and potential functional relevance of MP incorporation into starch–protein matrices [5].

In contrast, MS showed a highly refined composition characterised by a predominance of carbohydrates (87.23 g/100 g) and minimal contents of protein (0.88 g/100 g), fat (0.30 g/100 g) and ash (0.20 g/100 g). This composition is typical of refined starch ingredients commonly used as structural bases in extrusion processing, where starch plays a central role in melt formation, gelatinisation and expansion through the generation of a continuous viscoelastic matrix capable of retaining water vapour during flash‐off at the die exit. The literature on extrusion of starch–protein systems indicates that high starch fractions favour radial expansion and the formation of homogeneous cellular structures, particularly under conditions of low feed moisture content and elevated SME input, which enhance macromolecular transformation and bubble nucleation during extrusion [7].

SPC presented the highest protein content (37.92 g/100 g), together with intermediate ash content (5.86 g/100 g) and carbohydrate content (47.71 g/100 g), as well as a low fat fraction (0.34 g/100 g). Although the protein content may vary depending on the degree of industrial concentration and the basis of expression used, its high protein fraction is consistent with values reported for commercial concentrates (approximately 35%–70%), which are widely used to improve the nutritional quality of extruded products and to modify melt rheology through protein–starch interactions. During extrusion processing, soy proteins may contribute to the formation of a more cohesive matrix by promoting network development after denaturation; however, excessive protein incorporation may also reduce expansion capacity due to dilution of the continuous starch phase. Therefore, the combined use of MS, SPC and MPF provides a formulation framework in which the balance between starch continuity, protein network formation and fibre interference is expected to strongly influence the process parameters and the physical and techno‐functional properties of the extruded snacks evaluated in this study.

### 3.1. Effect of BT, MC, SPC/MS Ratio and MP Content on RT, TO and SME

RT values ranged from 26.12 to 35.96 s across the experimental domain (Table 5). Maximum RT was recorded at low MC (18.25 g/100 g), high SPC/MS (36.25 g/100 g) and high MP (21.25 g/100 g) (Tr14: 35.96 s), whereas minimum values occurred at higher MC (22.75 g/100 g), lower SPC/MS (28.75 g/100 g) and low MP (13.75 g/100 g) (Tr3: 26.12 s). The fitted quadratic model was significant (*p* < 0.05, *R* ^2^ = 0.77) with nonsignificant lack of fit (Table 3).

**Table 5:** Experimental data of extruded snack for response surface analysis.

Tr	BT (°C)	MC (g/100 g)	SPC/MS (g/100 g)	MP (g/100 g)	RT (g/min)	TO (Nm/s)	SME (J/g)	EI	AD (g/cm^3^)	H (H)	WAI (g/g)	WSI (%)	PH	*Δ*E
1	132.5	18.25	28.75	13.75	28.14	11.60	491.05	2.12	0.37	22.68	3.55	36.05	6.55	38.55
2	157.5	18.25	28.75	13.75	29.47	10.43	409.34	1.99	0.33	28.76	4.61	78.46	6.27	39.89
3	132.5	22.75	28.75	13.75	26.12	8.70	423.36	1.87	0.51	27.37	4.54	27.19	6.51	39.57
4	157.5	22.75	28.75	13.75	27.23	8.03	378.38	1.60	0.47	30.16	4.95	24.52	6.52	38.52
5	132.5	18.25	36.25	13.75	32.72	15.27	591.41	2.05	0.37	31.67	3.44	31.02	6.41	40.19
6	157.5	18.25	36.25	13.75	31.69	14.13	638.27	1.95	0.31	31.31	4.17	22.16	6.59	39.29
7	132.5	22.75	36.25	13.75	28.76	10.33	428.10	2.02	0.42	26.63	4.32	26.13	6.70	39.46
8	157.5	22.75	36.25	13.75	31.54	10.01	399.15	1.68	0.43	26.50	4.42	18.78	6.59	39.58
9	132.5	18.25	28.75	21.25	32.89	10.48	420.18	2.03	0.41	25.51	4.50	29.87	6.41	38.68
10	157.5	18.25	28.75	21.25	32.21	10.06	391.67	1.84	0.40	23.93	5.10	21.50	6.16	39.50
11	132.5	22.75	28.75	21.25	28.15	7.77	397.23	1.81	0.49	26.53	5.10	24.33	6.19	37.57
12	157.5	22.75	28.75	21.25	31.46	8.47	359.45	1.62	0.47	26.91	4.95	25.47	6.33	38.29
13	132.5	18.25	36.25	21.25	31.79	13.07	521.66	1.95	0.42	36.09	3.86	25.57	6.27	41.28
14	157.5	18.25	36.25	21.25	35.96	12.13	349.37	1.78	0.41	26.63	2.96	33.64	6.35	40.68
15	132.5	22.75	36.25	21.25	28.57	8.57	400.17	1.78	0.49	30.50	4.11	21.03	6.51	39.88
16	157.5	22.75	36.25	21.25	27.55	9.27	433.21	1.62	0.49	22.85	4.25	25.20	6.40	38.13
17	120.0	20.50	32.50	17.50	27.34	10.73	496.36	1.98	0.48	35.32	3.89	28.03	6.51	37.33
18	170.0	20.50	32.50	17.50	29.87	8.73	378.52	1.62	0.44	18.95	4.76	18.50	6.38	38.76
19	145.0	16.00	32.50	17.50	29.06	12.30	492.92	2.09	0.30	26.38	4.51	22.57	5.79	40.11
20	145.0	25.00	32.50	17.50	26.87	6.43	322.64	1.65	0.52	24.97	4.53	18.25	5.59	37.69
21	145.0	20.50	25.00	17.50	29.66	8.37	364.33	1.83	0.45	24.34	2.94	44.20	5.88	36.07
22	145.0	20.50	40.00	17.50	33.04	12.47	494.39	1.87	0.43	28.45	3.54	25.82	6.41	38.75
23	145.0	20.50	32.50	10.00	29.48	11.73	549.76	2.00	0.34	26.63	3.85	39.60	6.64	35.80
24	145.0	20.50	32.50	25.00	30.79	9.10	367.21	1.62	0.45	25.26	4.52	24.13	6.32	39.42
25	145.0	20.50	32.50	17.50	29.96	10.80	448.69	1.85	0.39	21.51	4.53	26.58	6.49	38.62
26	145.0	20.50	32.50	17.50	30.63	11.00	438.87	1.91	0.37	24.39	3.97	29.97	6.49	37.35
27	145.0	20.50	32.50	17.50	32.87	10.83	420.50	1.96	0.39	24.18	3.54	29.05	6.77	37.24
28	145.0	20.50	32.50	17.50	30.53	10.53	448.54	1.92	0.39	23.11	4.05	28.21	6.49	38.40
29	145.0	20.50	32.50	17.50	31.01	10.30	405.13	1.88	0.38	23.31	3.47	29.81	6.66	37.24
30	145.0	20.50	32.50	17.50	33.48	9.670	409.01	1.82	0.44	28.31	4.16	26.68	6.69	37.13

Abbreviations: *Δ*E, total colour difference; AD, apparent density; BT, barrel temperature; EI, expansion index; HA, hardness; MC, moisture content; MP, melon peel; MS, maize starch; RT, residence time; SPC, soy protein concentrate; TO, Torque; Tr, treatment; SME, specific mechanical energy; WAI, water adsorption index; WSI, water solubility index.

MC (B) and SPC/MS (C) were the dominant linear factors, with significant contributions from the quadratic term *B* ^2^ and the C × D interaction (SPC/MS × MP) (Table 3). MC exerted a negative linear effect on RT (*b*_2_ < 0; *p* < 0.05) with significant curvature (*b*_22_ < 0), whereas SPC/MS increased RT (*b*_3_ > 0; *p* < 0.05), consistent with the higher flow resistance of protein‐enriched melts. In this system, the starch–soy protein matrix combined with MP fibre generated a viscous blend whose RT was primarily governed by water availability and protein‐to‐starch balance.

**Figure 1:**
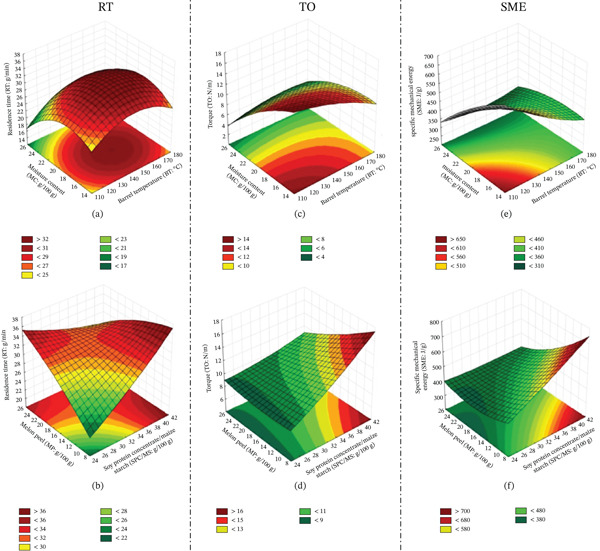
Response surface plot showing the effect of barrel temperature (BT), moisture content (MC), melon peel (MP) and soy protein concentrate/maize starch (SPC/MS) on: (a and b) residence time (RT); (c and d) torque (TO); and (e and f) specific mechanical energy (SME).

The response surfaces (Figure 1a) revealed maximum RT under intermediate‐to‐high BT with low MC, forming a dome‐shaped surface where the plasticising effect of moisture dominated over direct thermal input. The C × D interaction (Figure 1b) showed a compositionally dependent response: At low SPC/MS, increasing MP from 13.75 to 21.25 g/100 g raised RT (Tr1 vs. Tr9: 28.14 → 32.89 s), whereas at high SPC/MS, the same MP increase reduced RT (Tr5 vs. Tr13: 32.72 → 31.79 s; *b*_34_ < 0; *p* < 0.05). This pattern suggests that MP fibre behaves differently depending on the protein context: at low protein levels, MP fibre increased internal friction within the starch‐continuous matrix, prolonging residence; at higher protein levels, MP disrupted the starch–protein network cohesiveness, facilitating material transport and shortening RT.

TO ranged from approximately 8.4 to 10.1 N·m (Table 5), modulated primarily by MC and SPC/MS with additional effects from MP incorporation. The quadratic model was significant (*p* < 0.05, *R* ^2^ > 0.80) with nonsignificant lack of fit (Table 3). As with RT, MC exerted a negative linear effect on TO (*b*_2_ < 0; *p* < 0.05) and SPC/MS a positive one (*b*_3_ > 0; *p* < 0.05). In Figure 1c (TO vs. BT–MC), the pronounced downward slope along the MC axis confirms that moisture plasticisation was the dominant rheological driver in this system, whereas BT had no significant independent linear effect within the 120°C–170°C range evaluated. Figure 1d (TO vs. SPC/MS–MP) shows maximum TO under high SPC/MS and low MP, with the C × D interaction producing a slight TO reduction when MP increased at high protein levels a pattern consistent with the RT behaviour described above and attributable to partial disruption of the starch–protein network by MP fibre.

SME trends were consistent with changes in TO and RT, reflecting the interdependence of these parameters in the MP–soy–MS system (*R* ^2^ > 0.80; *p* < 0.05; nonsignificant lack of fit). MC had a significant negative linear effect on SME (*b*_2_ < 0; *p* < 0.05) and SPC/MS a positive one (*b*_3_ > 0; *p* < 0.05), with an additional C × D interaction and quadratic MC^2^ effect. Figure 1e (SME vs. MC/BT) illustrates maximum SME under low MC and low BT, whereas Figure 1f (SME vs. SPC/MS–MP) illustrates maximum SME under high SPC/MS and low MP. The composition‐dependent effect of MP on SME mirrored the pattern observed for RT and TO: at low SPC/MS, increasing MP raised SME, likely through increased friction at the starch‐melt interface; at high SPC/MS, it reduced SME, suggesting a partial loss of melt cohesiveness. BT had no significant linear effect on SME within the evaluated range, indicating that thermomechanical severity in this system was governed mainly by feed moisture and the protein‐to‐starch ratio rather than by barrel temperature alone.

Collectively, the coupled behaviour of RT, TO and SME in these MP extrudates confirms that the rheological state of the melt was determined by the balance between moisture‐driven plasticisation, protein‐induced structuring and fibre‐mediated interference. The compositionally dependent effect of MP modulated by the SPC/MS level represents a distinctive feature of by‐product‐enriched systems where nonstarch components alter matrix continuity and energy transfer depending on the formulation context [13, 14, 20–22].

### 3.2. Effect of BT, MC, SPC/MS Ratio and MP Content on AD

AD ranged from 0.30 to 0.52 g/cm^3^ (Table 5), with the minimum recorded at low MC (16 g/100 g) and high BT (145°C), and the maximum at MC = 25 g/100 g under equivalent BT, SPC/MS and MP conditions. This contrast directly reflects the inverse relationship between AD and EI observed across the experimental domain: conditions that limited expansion (high MC, high MP) consistently produced denser extrudates, whereas conditions favouring EI yielded lighter structures. The quadratic model (Table 3) showed excellent fit (*R* ^2^ = 0.94, *p* < 0.05, nonsignificant lack of fit), the highest among the responses evaluated, indicating that AD was particularly well described by the compositional and processing variables studied.

Significant effects included the linear terms BT (A), MC (B) and MP (D), the quadratic terms *A* ^2^ and *C* ^2^, and the interactions BD and CD (*p* < 0.05). MC increased AD (*b*_2_ = +0.038) and MP raised it further (*b*_4_ = +0.020), whereas BT showed a negative linear effect (*b*_1_ = −0.008) with positive curvature (*b*_11_ = +0.012). The interaction CD (*b*_34_ = +0.010) indicates that the compacting effect of MP was intensified when SPC/MS increased simultaneously, whereas BD (*b*_24_ = −0.009) suggests a partial compensatory effect between moisture and MP levels on density.

**Figure 2:**
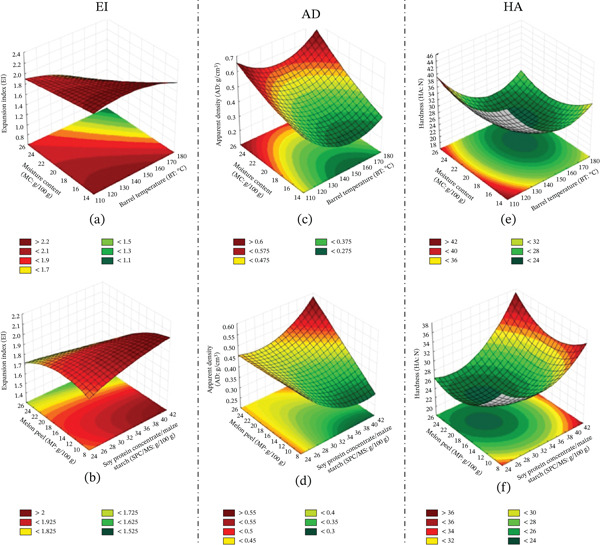
Response surface plot showing the effect of barrel temperature (BT), moisture content (MC), melon peel (MP) and soy protein concentrate/maize starch (SPC/MS) on: (a and b) expansion index (EI); (c and d) apparent density (AD); and (e and f) hardness (HA).

Figure 2c (AD vs. BT–MC) shows a pronounced gradient along the MC axis, with an intermediate BT region producing minimum density values. The nonmonotonic BT effect captured by the positive quadratic term b₁₁ is particularly relevant for this system: at lower BT, incomplete starch transformation limited melt development and expansion capacity, whereas at higher BT, thermally driven degradation of the starch–protein network reduced melt elasticity and promoted cell collapse, both outcomes resulting in higher AD. This is consistent with the negative BT effect on EI discussed in Section 3.2, and together these results define a narrow optimal BT window for achieving low‐density expanded structures in this MP–soy protein–MS matrix.

Figure 2d (AD vs. SPC/MS–MP) reveals that the simultaneous increase in SPC/MS and MP produced the highest AD values in the experimental domain. This combined effect reflects the dual contribution of MP fibre and soy protein to matrix rigidity: MP fibre introduces structural discontinuities that restrict bubble growth, whereas elevated SPC/MS increases the cohesiveness of the protein phase, collectively reinforcing cell walls at the expense of volumetric expansion. The BD interaction further indicates that this compacting tendency of MP was partially attenuated at higher MC levels, where increased moisture reduced melt cohesiveness and lessened the reinforcing effect of fibre on the extrudate structure.

Overall, AD in this system was primarily governed by the MC–BT balance, with MP acting as a systematic density‐raising component whose effect was modulated by protein content and moisture availability a behaviour that underscores the importance of compositional optimisation when incorporating MP as a functional ingredient in expanded extrudates.

[14, 21, 22].

### 3.3. Effect of BT, MC, SPC/MS Ratio and MP Content on HA

HA ranged from 18.95 to 36.09 N (Table 5). Maximum HA was recorded in Tr13 (BT = 132.5^°^C, MC = 18.25 g/100 g, SPC/MS = 36.25 g/100 g, MP = 21.25 g/100 g) and minimum in Tr18 (BT = 170^°^C, MC = 20.5 g/100 g, SPC/MS = 32.5 g/100 g, MP = 17.5 g/100 g). Notably, the softest extrudate did not correspond to the most expanded treatment, indicating that thermal severity rather than expansion alone was the primary determinant of fracture resistance in this system. The quadratic model was significant (*R* ^2^ = 0.72; *p* < 0.05; nonsignificant lack of fit; Table 3), with HA governed by the linear effect of BT and the interactions A × C (BT × SPC/MS), A × D (BT × MP) and B × C (MC × SPC/MS) (Table 4: *b*_1_ = −0.170, *b*_13_ = −0.148, *b*_14_ = −0.158, *b*_23_ = −0.173).

The dominance of interaction terms over individual linear effects is the defining structural feature of HA in this system: neither protein, fibre nor moisture independently determined HA; rather, their effects were conditional on barrel temperature. The negative linear effect of BT (*b*_1_ < 0) reflects thermally driven fragmentation of starch chains and partial dextrinisation at higher temperatures, which reduced cell wall strength and facilitated fracture consistent with the BT‐induced reduction in melt elasticity discussed for EI in Section 3.2. Figure 2e (HA vs. BT–MC) shows a clear decrease in HA along the BT axis, whereas MC had no significant independent linear effect, confirming that thermal severity not moisture plasticisation controlled fracture resistance in this MP–soy protein–MS matrix.

The interactions BT × SPC/MS and BT × MP (both negative) reveal that at higher BT, increasing either protein or MP fibre did not stiffen the extrudate and could in fact soften it further. This behaviour contrasts with the commonly reported hardening effect of fibre‐rich by‐products under moderate processing conditions and indicates that, at elevated temperatures, protein denaturation and starch phase degradation outweighed the structural reinforcement that MP fibre and soy protein would otherwise contribute through cell wall thickening. Figure 2f (HA vs. SPC/MS–MP) illustrates this nonadditive behaviour: at lower BT, increasing SPC/MS and MP together raised HA, whereas at higher BT the surface flattened or reversed, reflecting the thermal override of compositional effects.

The MC × SPC/MS interaction (*b*_23_ < 0) indicates that moisture conditioned the contribution of soy protein to texture, likely through competition for available water between starch gelatinisation and protein hydration, a balance that modified melt cohesiveness and the resulting cell wall integrity. This interaction explains why the effect of SPC/MS on HA was not significant as an independent term, yet emerged as a meaningful contributor through its coupling with both BT and MC.

Taken together, these results indicate that HA optimisation in MP‐enriched extrudates requires simultaneous control of BT, MC and formulation composition, since the structural outcome depends on coupled thermomechanical and compositional effects rather than on any single variable. In particular, moderate BT sufficient for starch transformation but below the threshold of network degradation combined with controlled SPC/MS and MP levels defines the operational window for achieving target texture without sacrificing the fibre‐enrichment potential of MP [21, 22].

### 3.4. Effect of BT, MC, SPC/MS Ratio and MP Content on the WAI

WAI ranged from 2.94 to 5.10 g/g (Table 5), with maximum values recorded at higher MC and MP levels (BT 132.5°C, MC 22.75 g/100 g, SPC/MS 28.75 g/100 g, MP 21.25 g/100 g) and minimum values at intermediate BT and MC with lower MP incorporation. The quadratic model showed adequate fit (*R* ^2^ 0.62, nonsignificant lack of fit; Table 3), with positive linear effects for BT (*b*_1_ > 0), MC (*b*_2_ > 0) and MP (*b*_4_ > 0) and a negative effect for SPC/MS (*b*_3_ < 0; Table 4).

### 3.5. Effect of BT, MC, SPC/MS Ratio and MP Content on EI

The EI of the extrudates ranged between 1.62 and 2.12 (Table 5), indicating a response typically sensitive to MC, BT, and the incorporation of nonstarch ingredients such as MP. For instance, higher EI values were observed under low‐to‐intermediate MC conditions (EI = 2.12 at BT = 132.5^°^C, MC = 18.25 g/100 g, SPC/MS = 28.75 g/100 g, MP = 13.75 g/100 g), whereas EI decreased to values close to 1.6 under formulation and processing conditions less favourable for expansion (Table 5). The second‐order model (Table 4) indicated that the significant linear terms (*p* < 0.05) affecting EI were BT (*b*_1_ = −0.035), MC (*b*_2_ = −0.040) and MP (*b*_4_ = −0.025) (in coded levels), whereas SPC/MS was not significant as an independent linear factor for EI (b_3_ ≈ 0.000), indicating that within the evaluated experimental domain the protein/starch balance did not directly control expansion capacity but rather modulated expansion behaviour through its interaction with moisture content (MC × SPC/MS).

The negative sign of b_2_ confirms that increasing moisture reduces expansion, which is consistent with extrusion theory: higher MC decreases melt viscosity, reduces shear stress and dissipative energy and limits the build‐up of pressure and the “flash‐off” of steam at the die exit, resulting in less expanded structures [5]. This pattern is clearly observed in the response surfaces shown in Figure 2a,b, where the regions of maximum EI are associated with relatively low MC levels [5, 23]. The negative coefficient b_4_ suggests that increasing MP tends to reduce EI, which is consistent with the behaviour of matrices containing higher fibre and ash contents. Fibre dilutes the starch available for gelatinisation and expansion, disrupts melt continuity and promotes denser microstructures, thereby reducing the ability of bubbles to grow during decompression [6, 24]. In other words, MP acts as a structural “filler” that may reinforce the cell wall of the extrudate, but at the cost of reduced volumetric expansion.

BT exerted a significant negative linear effect on EI (*b*_1_ = −0.035; *p* < 0.05) within the evaluated range (120°C–170°C). This finding appears counterintuitive given that barrel temperature is classically associated with enhanced starch gelatinisation and melt plasticisation; however, negative temperature–expansion relationships have been documented in starch‐based extrudate systems when operating above the threshold of complete gelatinisation [5, 23]. In the present system, the starch fraction was already exposed to sufficient thermomechanical energy at lower BT values as evidenced by the significant SME levels recorded across the experimental domain so that further increases in BT did not enhance gelatinisation degree but instead promoted excessive molecular degradation of amylose and amylopectin chains. High‐temperature depolymerisation reduces melt elasticity and storage modulus (G ^′^), which are critical for sustaining bubble wall integrity during postdie decompression: a less elastic melt cannot retain the vapour pressure generated at the die exit, leading to earlier cell rupture and coalescence, and ultimately to reduced volumetric expansion [23, 25]. Additionally, at elevated BT the faster evaporation of free water within the barrel may reduce the supersaturation conditions required for homogeneous bubble nucleation, further limiting EI [25]. In the context of the MP–soy protein–MS matrix evaluated here, where insoluble fibre from MP already disrupts melt continuity, high BT likely compounded this effect by further weakening the viscoelastic network of the starch‐rich phase. Consequently, the model identifies moderate BT combined with low MC as the most favourable region for expansion, a balance consistent with the need to maintain sufficient melt viscosity and elasticity for bubble stabilisation without inducing thermally driven structural degradation. The model identified a significant interaction affecting EI: MC × SPC/MS (*b*_23_ = +0.011, *p* < 0.05). This result is relevant because, although SPC/MS alone did not significantly affect EI (b_3_ ≈0), its positive interaction with MC suggests that the depressive effect of moisture may be partially attenuated when the SPC/MS ratio changes simultaneously. Mechanistically, this behaviour may be associated with rheological modifications of the melt. Soy protein may increase cohesiveness and elasticity under certain conditions, or modulate water distribution and network formation within the melt, thereby altering bubble nucleation and stability [23]. Accordingly, Figure 2a,b are consistent with a scenario in which maximum EI occurs under combinations of low MC and compositions that do not penalise the viscoelasticity of the system, whereas increasing MC shifts the response towards lower EI values unless the formulation (SPC/MS) partially compensates for this effect.

Consistent with the present findings, Ding et al. [5] reported that increasing moisture during the extrusion of expanded snacks reduces expansion due to lower effective shear and reduced pressure build‐up at the die exit, which agrees with the negative and significant b_2_ observed in this study. Likewise, the conceptual framework for expansion proposed by Alvarez‐Martinez et al. [23] indicates that expansion depends on the balance between melt viscosity and elasticity and the sudden generation of steam during decompression; conditions that deteriorate with high moisture levels and with formulations that dilute starch or disrupt the continuous matrix. In systems enriched with fibre‐rich by‐products, reductions in expansion are widely reported. Paraman et al. [3] discussed that the incorporation of agro‐industrial by‐products generally increases density and rigidity while reducing expansion, which is consistent with the negative effect of MP (b_4_). Similarly, Mazlan et al. [24] showed that increasing peel‐type ingredients and/or moisture tends to decrease expansion and modify process parameters (TO and moisture loss), supporting the interpretation of MP as a limiting factor for EI in expanded extrudates.

In addition to the individual effects of moisture, temperature and MP incorporation, the expansion behaviour observed in this study reflects the combined influence of melt rheology and mechanical energy transfer during extrusion processing. EI is strongly dependent on the ability of the starch‐rich phase to form a continuous viscoelastic matrix capable of retaining vapour pressure during sudden decompression at the die exit. Under the experimental conditions evaluated, reductions in EI at higher moisture contents and increasing MP levels are consistent with a decrease in melt viscosity and with structural discontinuities introduced by insoluble fibre particles, which interfere with bubble nucleation and growth. Furthermore, the trends observed for EI are coherent with the variations previously identified in TO and SME, since lower SME levels are generally associated with reduced macromolecular transformation and weaker melt elasticity, limiting the formation of highly expanded cellular structures. Similar relationships between fibre incorporation, melt rheology and expansion behaviour have been reported in extruded cereal‐based systems enriched with agro‐industrial by‐products, where the balance between starch continuity, protein interactions and fibre interference determine the final expansion capacity of the product [14, 21, 22].

**Figure 3:**
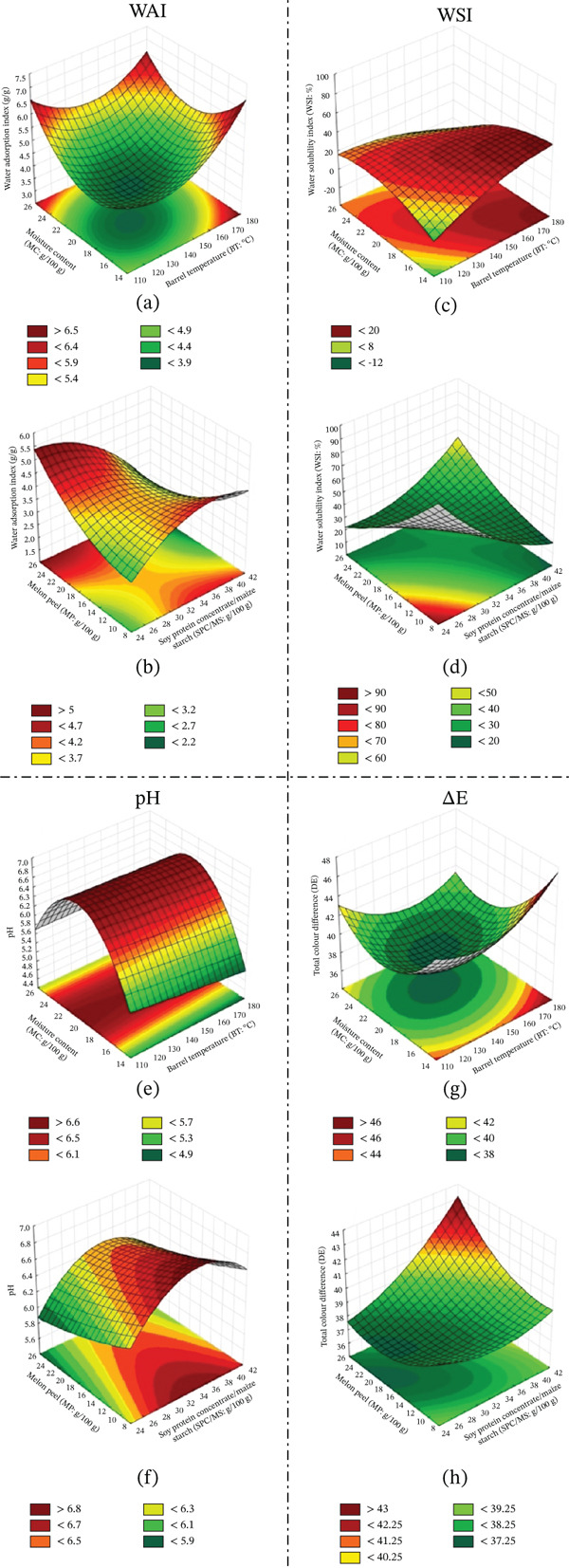
Response surface plot showing the effect of barrel temperature (BT), moisture content (MC), melon peel (MP) and soy protein concentrate/maize starch (SPC/MS) on: (a and b) water adsorption index (WAI), (c and d) water solubility index (WSI), (e and f) pH; and (g and h) total colour difference (ΔE).

The positive effect of BT and MC on WAI reflects enhanced starch gelatinisation and greater molecular mobility under higher thermomechanical severity, producing a more open, hydrophilic matrix. The response surface (Figure 3a, BT × MC) shows a bowl‐shaped curvature in which intermediate conditions minimise WAI, whereas extreme BT and MC values raise it, consistent with the nonlinear relationship between processing severity and starch transformation degree in this system.

The positive effect of MP on WAI is particularly relevant and merits specific attention. MP is characterised by a significant fraction of water‐soluble and water‐insoluble structural polysaccharides, primarily pectins, hemicelluloses and cellulose, that retain water through hydrogen bonding and capillary absorption within the fibre network [5, 26]. During extrusion, partial thermal and mechanical disruption of the cell wall releases and partially solubilises these polysaccharides, increasing their surface area and hydration accessibility in the extruded matrix. Pectic fractions, in particular, contribute to gel‐forming capacity upon hydration, whereas hemicellulosic components enhance bound‐water retention. Consequently, higher MP incorporation increased WAI not only by diluting the starch phase but by actively introducing hygroscopic structural polysaccharides that augmented the water‐binding capacity of the matrix, a mechanism distinct from starch gelatinisation and additive to it. This interpretation is supported by the positive MP contribution observed even under conditions where expansion was reduced (higher AD), indicating that fibre‐driven water retention operated independently of the degree of cellular development achieved at the die exit.

The negative effect of SPC/MS (*b*_3_ < 0) reflects competition between protein and starch for available water: higher protein fractions promote the formation of more compact starch–protein networks that restrict granule swelling and reduce overall hydration capacity, an effect modulated by MC as shown in Figure 3b (MC × SPC/MS interaction), where the depressive influence of SPC/MS on WAI became more pronounced at higher moisture levels.

Overall, WAI in this system was governed by the combined contributions of starch gelatinisation (BT, MC), fibre‐driven water binding (MP) and protein‐induced network compaction (SPC/MS). The dual role of MP reducing expansion while simultaneously enhancing water retention represents a functional trade‐off relevant to product design, where MP level must be optimised against the target balance between textural lightness and hydration capacity.

### 3.6. Effect of BT, MC, SPC/MS Ratio and MP Content on the WSI

The WSI exhibited a very wide range in the extrudates (18.25%–78.46%, Table 5), indicating that the degree of starch fragmentation/dextrinisation and the generation of soluble solids were highly sensitive to extrusion conditions and to the protein–starch ratio. The maximum WSI (78.46%) was obtained in Tr2 (BT = 157.5^°^C; MC = 18.25 g/100 g; SPC/MS = 28.75 g/100 g; MP = 13.75 g/100 g), whereas the minimum WSI (18.25%) was observed in Tr20 (BT = 145^°^C; MC = 25 g/100 g; SPC/MS = 32.5 g/100 g; MP = 17.5 g/100 g), experimentally confirming that increasing moisture markedly reduces the soluble fraction generated during the process.

The lack‐of‐fit test was not significant (*p* > 0.05), confirming the suitability of the fitted model for predicting WSI within the studied experimental range (Table 3).

The ANOVA results (Table 3) identified the linear terms of MC (B) and SPC/MS (C) as the dominant and significant effects (*p* < 0.05), whereas BT and MP did not explain significant variation by themselves within the evaluated range. This conclusion is reinforced by the model coefficients (Table 4), where MC (*b*_2_ = −0.328) and SPC/MS (*b*_3_ = −0.336) were negative and significant, indicating that increasing feed moisture and raising the relative protein proportion reduce the solubility of the extrudate. Mechanistically, this pattern is consistent with the extrusion behaviour of starch–protein matrices: increasing MC plasticises the melt, lowers its viscosity and reduces the effective shear stress, thereby limiting starch chain rupture and the formation of soluble dextrins, which ultimately leads to lower WSI values [5, 27].

Similarly, increasing the SPC/MS ratio tends to decrease WSI due to starch dilution and the development of protein–starch networks or aggregates that limit degradation and the release of soluble solids. This effect has been reported for maize–soy blends and other extruded protein–starch systems [7, 28]. The response surfaces corroborate this rheological–compositional control. In Figure 3c (WSI vs. BT–MC), a pronounced gradient dominated by moisture is observed, with maximum WSI occurring at low MC and progressively decreasing as MC increases, whereas the effect of BT is secondary (consistent with a near‐zero and nonsignificant b₁ coefficient). In Figure 3d (WSI vs. SPC/MS–MP), the surface shows a decrease in WSI with increasing SPC/MS, whereas MP plays a comparatively minor role in the linear term (nonsignificant b_4_). However, the positive interaction coefficient (b_34_) suggests that the effect of protein may be modified in the presence of MP, possibly due to changes in internal friction and microstructure that influence the release of soluble fractions [24].

Overall, WSI in this system is primarily governed by the MC–SPC/MS combination, where low MC and lower SPC/MS favour higher solubility (greater “mechanical cooking” and dextrinisation), whereas high MC and higher SPC/MS promote less soluble extrudates, which are likely to be structurally more stable and exhibit lower release of soluble solids. This behaviour is particularly relevant for controlling rehydration properties, mouthfeel viscosity and the functional performance of the final extruded product.

These variations in WSI are also consistent with the thermomechanical severity previously identified through the changes observed in SME and expansion behaviour. In extrusion systems based on starch‐rich matrices, higher WSI values are generally associated with increased molecular degradation of starch and the formation of low‐molecular‐weight dextrins generated under conditions of higher shear stress and lower feed moisture. Conversely, lower WSI values obtained at higher moisture levels reflect reduced mechanical energy transfer and limited starch fragmentation during processing. In addition, the incorporation of MP fibre may contribute to restricting the release of soluble solids by reinforcing the structural matrix and reducing starch availability for dextrinisation. Therefore, the combined effects of moisture plasticisation, protein–starch interactions and fibre incorporation determine the balance between gelatinisation and molecular degradation, which ultimately governs the solubility behaviour of the extruded products. Similar relationships between extrusion severity, starch transformation and soluble fraction development have been reported in cereal‐based extrudates enriched with plant coproducts [5, 21, 22].

### 3.7. Effect of BT, MC, SPC/MS Ratio and MP Content on pH

The pH of the extrudates ranged from 5.59 to 6.77 (Table 5), indicating that even with the incorporation of MP, the products remained within a slightly acidic to nearly neutral range. This range is consistent with the physicochemical stability of the starch–protein matrix and does not represent a concern for product safety or quality. From a practical standpoint, the moderate decrease in pH at higher MP levels may influence flavour perception (slight sourness) and could contribute to the preservation of phenolic compounds and antioxidant stability, given that acidic conditions generally protect these bioactive components from oxidative degradation.

The quadratic model fitted for pH showed an intermediate level of fit (*R* ^2^ = 0.73; Table 3), with a significant linear effect of MP content and a significant quadratic effect of MC, whereas BT, the SPC/MS ratio and the interaction terms were not significant (*p* < 0.05). The lack‐of‐fit test was not significant (*p* > 0.05), indicating that the model adequately represented the experimental data.

This pattern suggests that pH variation was governed primarily by the composition of the mixture and, to a lesser extent, by a curvilinear response to feed moisture, rather than by direct changes in the thermal severity of the process.

The lowest pH values were observed under extreme moisture conditions, particularly at 25 g/100 g MC (pH = 5.59) and at 16 g/100 g (pH = 5.79), whereas the highest values occurred under intermediate moisture conditions, around the central point of the design, where the pH reached up to 6.77. This behaviour is illustrated in Figure 3e, where the response surface shows a pronounced curvature with respect to MC and a comparatively smaller effect of BT. This pattern may be interpreted as a consequence of the dual role of moisture during extrusion: on one hand, very low MC promotes a higher effective concentration of solutes and more intense matrix transformations; on the other hand, high MC increases hydration and the release of low–molecular‐weight compounds present in the fibrous fraction, including organic acids and phenolic compounds, which may shift the pH towards lower values. In contrast, at intermediate moisture levels an equilibrium is established between aqueous dispersion, dilution of acidic compounds and starch–protein matrix reorganisation, explaining the maximum observed in the central region of the surface.

Figure 3f further confirms that the incorporation of MP tended to decrease pH, particularly as MP increased towards 25 g/100 g. Although the surface visually suggests that a higher SPC/MS level might partially buffer this decrease, the ANOVA indicates that the linear effect of SPC/MS was not significant for pH; therefore, this trend should be interpreted with caution. This behaviour is consistent with the composition of the by‐product: MP contains dietary fibre, minerals (ash) and a significant fraction of phenolic compounds and organic acids, components capable of modifying the apparent acidity of the system, particularly when incorporated at high levels [2]. Thus, the decrease in pH with increasing MP can be interpreted primarily as a compositional effect rather than as a direct consequence of thermal treatment itself.

These findings are consistent with reports in other extruded systems, where feed moisture and fibrous composition exert a more consistent influence on physicochemical quality than temperature when the process occurs under short RTs and high shear conditions [26, 29]. Similarly, in extruded blends rich in nonstarch ingredients, moisture often modulates the extent of gelatinisation, solubilisation and dispersion of hydrophilic components, indirectly affecting properties such as pH, WAI and WSI [20, 30].

These pH variations can also be interpreted in relation to the chemical transformations occurring during extrusion processing, where short RTs combined with high shear conditions generally limit extensive acidification reactions but may promote the release of low–molecular‐weight compounds from plant cell wall structures. In fibre‐enriched extrusion systems, partial depolymerisation of hemicelluloses and pectic substances, together with the mobilisation of phenolic acids naturally present in fruit by‐products, may contribute to moderate decreases in pH without producing drastic changes in the overall acid–base balance of the matrix. Furthermore, the relatively stable pH range observed in the present study is consistent with typical cereal–protein extrudates, in which starch gelatinisation and protein denaturation occur without substantial formation of acidic degradation products. Similar behaviour has been reported in extruded formulations enriched with fruit and vegetable coproducts, where compositional factors rather than thermal severity are the main determinants of pH variation [5, 21, 22].

### 3.8. Effect of BT, MC, SPC/MS Ratio and MP Content on ΔE

ΔE ranged from 35.80 to 41.28 (Table 5), indicating substantial colour change across all treatments relative to the reference, with minimum values at intermediate processing conditions (BT≈145°C, MC≈20 g/100 g, SPC/MS≈30 g/100 g, MP≈18 g/100 g) and maximum at high thermal severity and low moisture (BT≈180°C, MC≈14 g/100 g, SPC/MS≈38 g/100 g, MP≈26 g/100 g). The quadratic model showed adequate fit with nonsignificant lack of fit (Table 3). Significant terms were the linear effect of SPC/MS (C; *b > 0*) and the quadratic term of MC (*B* ^2^; *b* > 0), whereas BT and MP were not significant as independent linear factors.

The positive SPC/MS effect reflects greater availability of free amino groups from soy protein for Maillard condensation with reducing sugars, intensifying melanoidin formation under thermomechanical conditions. The quadratic MC effect produced a basin‐shaped surface (Figure 3g) in which intermediate moisture minimised *Δ*E: at low MC, reduced melt lubrication increased SME and localised heating, accelerating browning; at high MC, excess water may have promoted alternative degradation pathways or enhanced reactant mobility, sustaining colour development through a different route.

The combined effect of MP and SPC/MS on ΔE (Figure 3h) is particularly relevant for this system. MP contributes phenolic compounds including flavonoids and hydroxycinnamic acid derivatives characteristic of *C. melo* by‐products that undergo thermal oxidation and participate in secondary browning pathways during extrusion, intensifying colour development beyond that expected from the starch–protein matrix alone [31]. At high SPC/MS combined with high MP, the convergence of Maillard substrates (amino groups from protein, reducing sugars from starch hydrolysis) and oxidisable phenolic substrates from MP produced the highest ΔE values in the experimental domain. This additive browning mechanism, Maillard plus phenolic oxidation, is a distinctive feature of fruit by‐product‐enriched extrudates and explains why ΔE responded more strongly to formulation composition than to BT or MC individually [5, 21].

From a product design perspective, the moderate ΔE range observed (35.80–41.28) suggests that colour uniformity can be maintained across a wide operational window provided that SPC/MS and MP levels are controlled simultaneously, with intermediate MC offering an additional lever to minimise undesired browning intensity.

## 4. Conclusion

The present study demonstrated that feed moisture content and mixture composition are the main factors controlling mechanical energy transfer, melt rheology and the structural development of extruded snacks enriched with MP flour. Increasing MC significantly reduced RT, TO and SME, whereas higher SPC/MS ratios increased TO and energy input; MP incorporation consistently reduced EI and increased AD and HA through fibre‐induced dilution of the continuous starch phase and disruption of melt viscoelasticity. The quadratic RSM models fitted for all response variables showed adequate predictive capability (*R* ^2^ = 0.62–0.94, nonsignificant lack of fit), and model validation confirmed agreement between predicted and observed values within the evaluated experimental domain. The interaction effects identified through RSM highlight that MP incorporation cannot be optimised independently of feed moisture and protein–starch ratio, as the final product structure depends on coupled thermomechanical and compositional effects. The results confirm the technological potential of MP as a fibre‐rich ingredient for extrusion‐based snack development within a circular economy framework, with evaluated processing conditions falling within typical operating ranges for cereal‐based systems; however, further studies addressing nutritional profiling, antioxidant activity and consumer acceptance are required to fully support the development of functional extruded snack formulations based on this by‐product.

## Funding

No funding was received for this manuscript.

## Conflicts of Interest

The authors declare no conflicts of interest.

## Data Availability

The data that support the findings of this study are available from the corresponding author upon reasonable request.
